# The Dual PDE7-GSK3β Inhibitor, VP3.15, as Neuroprotective Disease-Modifying Treatment in a Model of Primary Progressive Multiple Sclerosis

**DOI:** 10.3390/ijms232214378

**Published:** 2022-11-19

**Authors:** Rocio Benítez-Fernández, Carmen Gil, Carmen Guaza, Leyre Mestre, Ana Martínez

**Affiliations:** 1 Centro de Investigaciones Biológicas Margarita Salas-CSIC, Ramiro de Maeztu 9, 28040 Madrid, Spain; 2 Instituto Cajal-CSIC, Doctor Arce 37, 28002 Madrid, Spain; 3 Centro de Investigación Biomédica en Red en Enfermedades Neurodegenerativas (CIBERNED), Instituto de Salud Carlos III, 28029Madrid, Spain

**Keywords:** multiple sclerosis, PDE7, GSK3β, remyelination, TMEV-IDD, PPMS

## Abstract

Multiple sclerosis (MS) is a chronic, inflammatory, autoimmune and degenerative disease with axonal damage and demyelination as its main features. Its dual neurological and autoimmune nature makes it a disease that is difficult to treat. Treatments that simultaneously stop the immune response while protecting and repairing the nervous system are urgent. That is of utmost importance for the primary progressive multiple sclerosis (PPMS), a rare and severe variant of MS, characterized by worsening neurological function from the onset of symptoms. In this sense, inhibitors of glycogen synthase kinase 3β (GSK3β) and phosphodiesterase 7 (PDE7) have recently shown great therapeutic potential for the treatment of demyelinating diseases. Here we investigated a dual inhibitor of these two targets, the small molecule VP3.15, in a preclinical model, which resembles primary-progressive MS (PPMS), the Theiler’s mouse encephalomyelitis virus-induced demyelinated disease (TMEV-IDD). In our study, VP3.15 ameliorates the disease course improving motor deficits of infected mice. Chronic treatment with VP3.15 also showed significant efficacy in the immunomodulation process, as well as in the proliferation and differentiation of oligodendroglial precursors, improving the preservation of myelin and axonal integrity. Therefore, our results support a treatment with the safe VP3.15 as an integrative therapeutic strategy for the treatment of PPMS.

## 1. Introduction

Multiple sclerosis (MS) is the most frequent autoimmune demyelinating disease of the central nervous system (CNS) affecting about 2.3 million people around the world [1]. Glial cell pathology (especially oligodendrocytes and their precursors), inflammatory processes, demyelination and axonal damage characterize it [2]. Among the MS variants, primary progressive multiple sclerosis (PPMS) constitutes a severe and rare group that involves much less inflammation of the type seen in relapsing MS. As a result, people with PPMS tend to have fewer brain lesions (also called plaques) than people with relapsing MS and the lesions tend to contain fewer inflammatory cells. Furthermore, patients of PPMS tend to have more lesions in the spinal cord than in the brain [3]. Therefore, it is of pivotal importance to restore lost myelin, and thus, reduce the neurological dysfunction by, for example, modulating the inflammatory response, promoting an effective remyelination and protecting oligodendrocytes. Current available treatments are almost exclusively immunomodulatory, focused on reducing the inflammation of the active-demyelinating plaque, so they cannot be considered curative and their effects are minimal in PPMS patients. Indeed, the direct or indirect contribution of immunomodulatory therapies over repairing/regenerative mechanisms remains elusive.

Currently, the experimental autoimmune encephalomyelitis (EAE) model is the most widely used model for the development of new treatments for MS [4]. Among its main advantages are the neuropathological, clinical and immunological similarities with MS. Moreover, it is a fast model with high reproducibility due to the fact that the immunogenic inducer is known [5]. However, these properties may turn in a disadvantage since the main characteristic of MS is the unknown etiology and relapsing events are not present in PPMS. Another experimental MS animal model, the Theiler’s Murine Encephalomyelitis Virus-Induced Demyelinating Disease (TMEV-IDD), is a viral model that mimics human MS in a variety of aspects that include chronic inflammation involving CD4^+^ and CD8^+^ T cells, B cells and myeloid cells; sex influenced disease course and symptomatology such as gait alterations and incontinence. Histopathology signs of axonal degeneration and spinal cord atrophy during the chronic phase of TMEV-IDD closely resemble the primary and progressive clinical forms of MS disease [6,7]. Therefore, TMEV-IDD is an excellent pharmacological tool to discover new treatments with innovative mechanisms of action for the primary progressive forms of MS (PPMS) [8,9]. PPMS is an aggressive and neurodegenerative form of MS without specific treatment, thus discovery of new drugs is an urgent need.

The three main events that occur in all neurodegenerative diseases are excitotoxicity, neuroinflammation and oxidative stress. For this reason, the study of new therapeutic agents will be focused on interfering in these processes. On this line, the inhibition of the glycogen synthase kinase 3β (GSK3β) favors numerous neuronal processes in embryonic and adult development such as neuronal morphogenesis, axonal growth, proliferation, cell differentiation, cell survival and neuronal polarization [10]. Currently, GSK3β inhibitors have been proposed as possible neuroprotective drugs for the treatment of retinitis pigmentosa [11]; as modulators of cytosolic accumulation of TDP-43 during the cellular stress [12]; as a potential treatment for Parkinson’s disease [13]; as regulators of Th1 cells in EAE mice model [14] or as a neurorepair treatment in MS [15]. Additionally, the study of phosphodiesterase 7 (PDE7) as a therapeutic target arose both due to its high specificity for cAMP and its high expression in the brain, thus avoiding possible adverse effects [16]. PDE7 inhibitors have been studied as possible inhibitors of T lymphocyte proliferation [17] and promoters of oligodendrocyte survival and proliferation, thus showing a high potential for the treatment of MS [18]; additionally, they have been studied as neuroprotective drugs for the treatment of Parkinson’s disease [19]. With the aim of obtaining more powerful inhibitors capable not only of modulating the immune response, but of promoting the differentiation and proliferation of neuronal cells, the synthesis of multi-target compounds for GSK3β and PDE7 was proposed for the treatment of neurodegenerative and demyelinating diseases [20]. Multi-target drugs have been proposed as a therapeutic alternative for diseases of unknown and multifactorial etiology since they are individual chemical entities capable of modulating different targets simultaneously [21]. Within the group of dual inhibitors against GSK3β and PDE7, the most promising compound was VP3.15 thanks to its neuroprotective and immunomodulatory function in primary astrocyte cultures, its ability to cross the blood-brain barrier, high safety profile and good pharmacodynamic and pharmacokinetic properties after oral and intraperitoneal administration [22]. Furthermore, VP3.15 has been described as promoter of remyelination in vitro in both mouse and human oligodendrocyte progenitor cells (OPC) cultures, and in vivo in a cuprizone model, suitable for the study of remyelination regardless of the immune response [15]. The combination of GSK3β and PDE7 inhibition can synergistically activate beneficial anti-inflammatory and pro-remyelinating pathways with high therapeutic potential.

Here, we assessed the therapeutic potential of VP3.15 on the Theiler’s virus model of progressive MS. The follow-up of the neurological deficits, axonal damage, leukocyte infiltration and pro-inflammatory mediators was made with the aim of showing the therapeutic potential of this dual inhibitor for this unmet and severe disease

## 2. Results

### 2.1. The PDE7/GSK3 Dual Inhibitor VP3.15 Ameliorates Motor Function of TMEV-IDD

**Figure 1:**
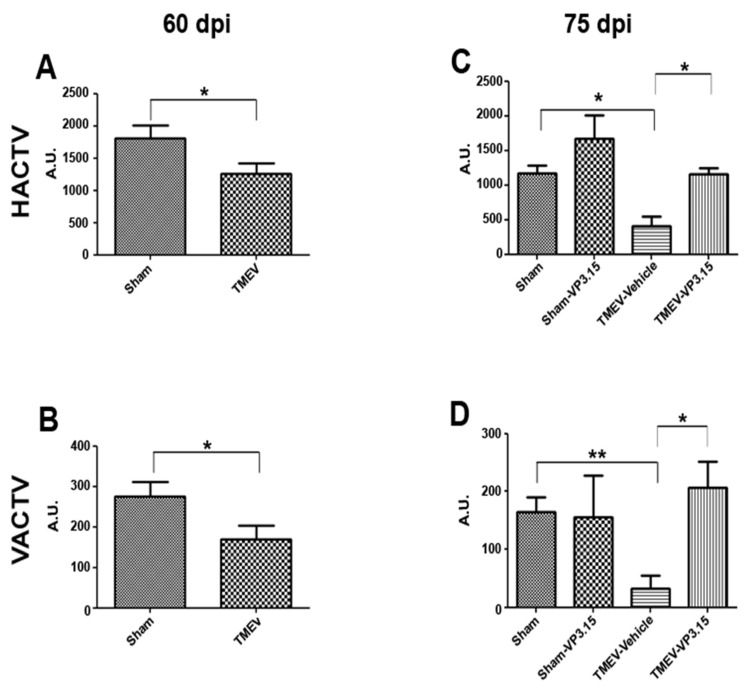
The treatment with VP3.15 delays TMEV-IDD progression. TMEV-infected mice were treated with VP3.15 (10 mg/kg) or appropriate vehicle at 60 days post infection for 15 consecutive days. The motor function was analyzed at days 60 (**A**,**B**) and 75 post infection (**C**,**D**) in an activity cage, including the horizontal activity (HACTV) and the vertical activity (VACTV) for 10 min. Student’s *t*-test (**A**,**B**): * *p* < 0.05; Sham vs. TMEV; ANOVA (**C**,**D**) * *p* < 0.05; ** *p* < 0.01 Sham-vehicle vs. TMEV-vehicle. The sample size was Sham = 11, TMEV = 14, Sham-Vehicle = 7, Sham-VP3.15 = 4, TMEV-Vehicle = 7, TMEV-VP3.15 = 7.

Intrathecal infection of a susceptible strain of mice with Theiler’s virus produces motor disability that arises after a latency period that varies depending on the viral strain used. Previous studies from our group have reported a latency of 60–70 days after Daniel strain inoculation [8]. Here, motor dysfunction was witnessed through the decrease in the horizontal (HACTV) and vertical activity (VACTV) displayed by TMEV-infected mice at 60 days post infection (Figure 1A,B). The treatment with VP3.15 (10 mg/kg) for 15 consecutive days significantly counteracts the motor deficits observed both horizontal and vertical activities (Figure 1C and 1D, respectively).

### 2.2. VP3.15 Limits Microglial Activation and Lymphocyte Infiltration in the Spinal Cord of TMEV Infected Mice

**Figure 2:**
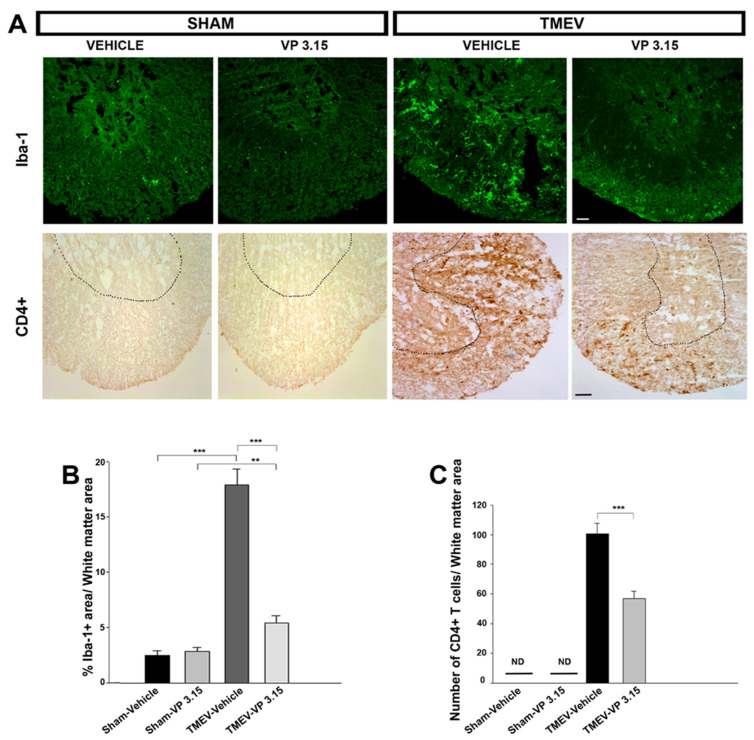
Treatment with VP3.15 significantly reduces Iba-1 staining and infiltration of CD4^+^ T lymphocytes. (**A**): Panoramic views of the spinal cord labeled for Iba-1^+^ (green) and stained to visualize CD4^+^ T-cells. (**B**,**C**): Quantification analysis of percentage of area stained by Iba-1^+^ (**B**) and number of CD4^+^ T-cells of white matter area (**C**). Scale bar represents 50 µm (Iba-1^+^) and 200 µm (CD4^+^T cells). Abbreviations: ND = non-detected. The results of the Student’s *t* test are expressed as ** *p* < 0.01; *** *p* < 0.001. The results of the One-Way Anova indicated significant differences (*p* < 0.05) in the percentage of area marked with Iba-1. (at least 5 slices/mice). The sample size was Sham-Vehicle = 7, Sham-VP3.15 = 4, TMEV-Vehicle = 7, TMEV-VP3.15 = 7.

Immunohistochemical analysis of the spinal cord of TMEV-infected mice revealed that the treatment with VP3.15 induced a significant attenuation of neuroinflammation (Figure 2A). TMEV-IDD is characterized by microglial activation in the spinal cord, and cytotoxic factors produced by activated microglia have been associated with ongoing demyelination. As shown in Figure 2B, TMEV-infected mice showed an intense microglial staining (*p* < 0.001 vs. Sham mice) while VP3.15 treated mice showed similar levels to sham mice (*p* < 0.001 vs. TMEV). In the same line, the presence of infiltrated CD4^+^ T cells in the spinal cord of TMEV-mice was significantly decreased in those that were treated with VP3.15 (*p* < 0.001 vs. TMEV) (Figure 2C).

### 2.3. The Remyelinating Role of VP3.15 and Its Effect in the Integrity of Axons of TMEV Mice

**Figure 3:**
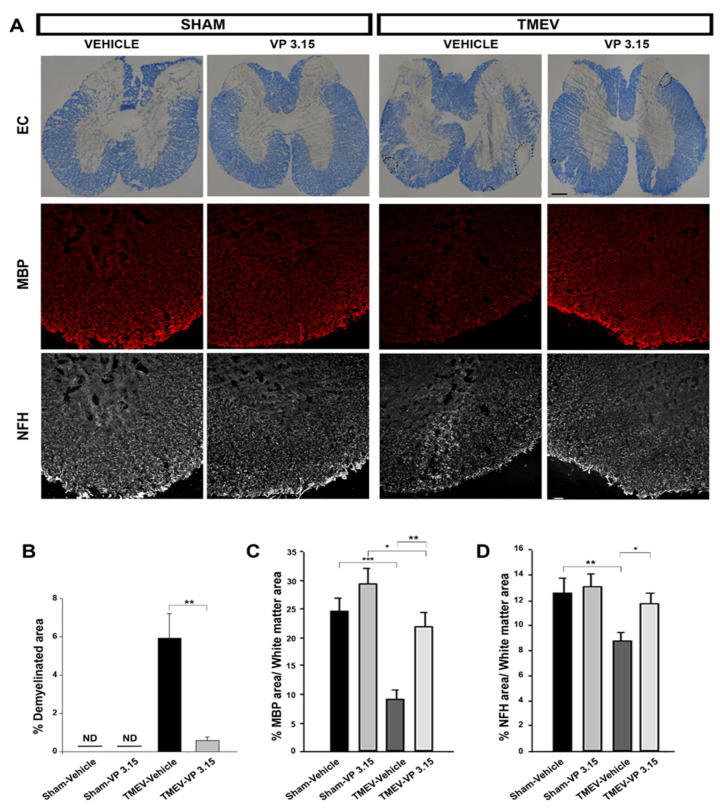
The spinal cord of VP3.15-treated mice presented a lower level of demyelination and axonal damage. (**A**): Detailed views of the spinal cords of vehicle and VP3.15-treated mice stained with eriochrome cyanine and labeled for MBP (red) and NFH (grey). (**B**): Graph showing a decreased percentage of demyelinated area with respect to the white area in the VP3.15-treated mice. The dashed line delimits the demyelinated area. (**C**,**D**): Histograms of the quantification showing a significant increase in the percentage of both MBP^+^ and NFH^+^ area in the VP3.15-treated compared to the vehicle-treated mice. Scale bar represents 200 µm (EC) and 50 µm. Abbreviations: EC = eriochrome cyanine; MBP = myelin basic protein; NFH = neurofilament heavy; ND = non-detected. The statistical analysis was performed using the Student’s *t* test, with the following values: * *p* < 0.05; ** *p* < 0.01; *** *p* < 0.001. The sample size was Sham-Vehicle = 7, Sham-VP3.15 = 4, TMEV-Vehicle = 7, TMEV-VP3.15 = 7.

As mice improve their motor activity after VP3.15, we wondered whether this attenuation of disability could be related to better conservation of myelin and/or an improvement of axonal integrity (Figure 3A). The demyelination visualized by eriochrome cyanine staining showed that the percentage of demyelinated area in the spinal cord of TMEV-infected mice was significantly reduced in TMEV-mice treated with VP3.15 (Figure 3B). 

In addition, an analysis of the myelin status was carried out by the myelin basic protein or MBP labeling, where the TMEV-vehicle mice presented a significant loss of myelin in comparison with their Sham group. In contrast, VP3.15 treatment significantly counteracted the myelin loss observed in TMEV-vehicle mice (Figure 2C). Finally, axonal damage was analyzed by the anti-neurofilament NFH antibody. TMEV mice showed a significant reduction of the percentage of NFH stained area relative to Sham mice. The group of animals treated with VP3.15 practically recovered the levels of NFH observed in Sham mice (Figure 2D).

### 2.4. VP3.15 Promotes the Presence of Mature Oligodendrocytes in Spinal Cord

**Figure 4:**
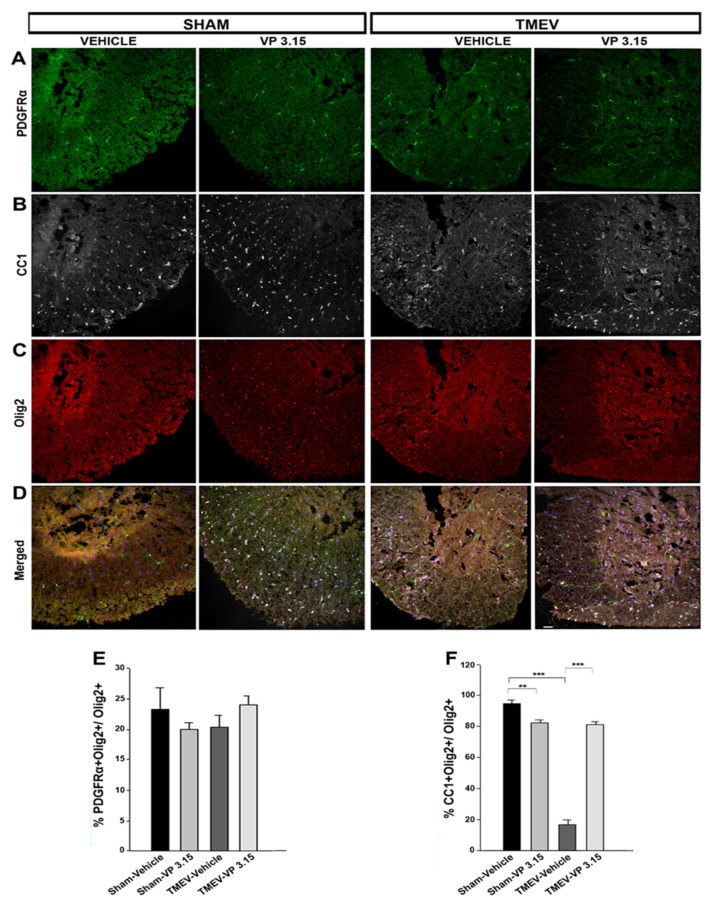
The VP3.15 promotes the presence of mature oligodendrocytes in spinal cord. (**A**–**D**): Detailed views of the spinal cords of vehicle and VP3.15-treated mice, labeled for PDGFRα (green), Olig2 (red), CC1 (grey) and merged includes nuclei (Hoechst; blue). Scale bar represents 50 µm. (**E**,**F**): Graphs are showing the significant increase in the percentage of PDGFRα^+^Olig2^+^ cells (E) and Olig2^+^CC1^+^ cells (**F**) after the treatment with VP3.15 compared to the vehicle. The statistical analysis was performed using the Student’s *t* test, with the following values: ** *p* < 0.01 Sham versus TMEV-Vehicle; *** *p* < 0.001 TMEV-Vehicle versus TMEV-VP3.15. The sample size was Sham-Vehicle = 7, Sham-VP3.15 = 4, TMEV-Vehicle = 7, TMEV-VP3.15 = 7.

Next, we inquired whether the remyelinating and the neuroprotection of axons could be related to changes in the oligodendrocyte’s lineage. In the TMEV infection, the recruitment of OPCs in the lesion areas appears to be insufficient and their differentiation capacity is affected since spontaneous remyelination is incomplete [7,23]. In the study of the oligodendrocyte lineage, we labeled OPCs with PDGFRα (Figure 4A), mature cells with CC1 (Figure 4B), all cells of the lineage with Olig2 (Figure 4C) and then merged staining (Figure 4D). There is no significant change in the number of precursor cells, labeled as PDGFRα+ cells (Figure 4E). Nevertheless, a significant increase in the number of mature cells, identified as CC1^+^ cells, was observed in TMEV-mice treated with VP3.15 (Figure 4F). These in vivo data suggest that our treatment with VP3.15 in the TMEV-IDD model, although it does not generate proliferation of OPCs, does stimulate their differentiation towards myelinating phenotypes, which is consistent with the previous results.

## 3. Discussion

Approximately 10% of patients with MS are affected by the “primary-progressive” variant, characterized by a chronic and progressive increase in neurological involvement without intermediate periods of remission. It is the most disabling form of the disease marked by the accumulation of irreversible disability caused by deep axonal damage, although it is characterized by having less inflammatory activity than the “relapsing-remitting” variant. In fact, now PPMS belongs to the progressive forms of MS, including the secondary progressive MS (SP-MS) [24], expanding the interest of new therapies. Currently, there is only one drug on the market for this form of MS, ocrelizumab, a recombinant humanized monoclonal antibody that acts selectively against CD20+ lymphocytes [25]. The exact mechanism by which ocrelizumab exerts its clinical effects has not been completely elucidated, and it has not been possible to demonstrate its actions on the repair/regeneration mechanisms of myelin [26].

Our lab has wide expertise using the viral TMEV model as a preclinical tool for new therapeutic compound discovery [8,9,27,28]. Our well-established protocol indicates that approximately 60–70 days post-infection mice start to show motor deficits. At this time, 15 days of treatment are usually enough to visualize the therapeutic effects of the studied compounds. Previously, the therapeutic potential of a selective PDE7 inhibitor (TC3.6) in the TMEV-IDD model was evaluated. The inhibitor was administrated both in the presymptomatic phase and once the disease was established, which makes it possible to determine whether the compound is effective both in preventing symptoms and reducing them. Inhibition of PDE7 by the compound improved motor function in the TMEV-IDD model in both administration windows [28]. In that time, neuroinflammation parameters were evaluated showing a clear reduction on them. Regarding oligodengrogenesis, it was only studied in vitro using OPCs from rats, and in comparison with GSK3β and dual GSK3β/PDE7 inhibitors [15]. Results obtained pointed that the concomitant dual activity favored OPC differentiation, so these compounds were selected for the next assays.

Thus, in this study, we proposed for the first time the dual inhibition of GSK3β and PDE7 as a therapy for PPMS by analyzing the neuroprotective, anti-inflammatory and neurorepairing activity of VP3.15. The compound was evaluated only after symptoms appeared, to mimic the time when treatment was started in MS patients. Our studies show that treatment with VP3.15 for 15 days made it possible to recover almost all the motor deficits observed in TMEV mice. VP3.15 proved to be a promising potential treatment of this MS variant, unlike fingolimod, which did not show any neuroprotective activity in the TMEV model. This could be due not only to the time chosen for its administration, but also to a lack of involvement of this drug in the repair mechanisms [29].

As we have seen previously, CD4^+^ T lymphocyte infiltrates and microglial activation play an important role in the pathogenesis and course of TMEV disease. Here, we investigated the anti-inflammatory role of VP3.15, first by taking into account that increased levels of cAMP by inhibiting PDE7 significantly attenuated the inflammatory response both by reducing lymphocyte infiltration and microglial activation [30] and second, that the modulation of the NF-κB pathway by inhibiting GSK3β, decreases the inflammatory response [31]. Indeed, the results of the present study affirm that this dual inhibitor was able to decrease both microglial activation and infiltration of CD4^+^ T lymphocytes. We suggest that the joint inhibition of PDE7 and GSK3β favors a neuroprotective process that could decrease chronic demyelination and even promote myelin repair mechanisms. This was contrasted with the study of myelin preservation, where it was observed that the VP3.15 inhibitor actually promotes greater myelin conservation by reducing the number of demyelinated areas in the eriochromocyanin staining and counteracting the loss of MBP. Axonal damage begins in the presymptomatic phase of the TMEV-IDD model and is exacerbated in the chronic phase due to the autoimmune response [32]. In our previous study, PDE7 inhibition improved axonal integrity [28]; the present study deserves special interest as the treatment with VP3.15 in TMEV animals allowed them to recover levels of axonal integrity similar to control mice. Probably, the simultaneous inhibition of PDE7 and GSK3β increases the preservation of axonal integrity. Regarding the behavior of cells of oligodendroglia lineage, our data reveals that the number of OPCs in the spinal cord of infected mice did not change independently of the treatment with VP3.15. This observation suggests VP3.15 is not affecting to the proliferation of OPCs in this model, as was previously observed in vitro [15]. Importantly, TMEV infection affects the differentiation of OPCs, showing a highly significant decrease in mature oligodendrocytes with myelinating capacity [23], something that in our case, was seen counteracted with by the administration of VP3.15.

On the whole, the results of the present study allow us to affirm that treatment with a dual inhibitor of GSK3β and PDE7 in a “primary-progressive” preclinic MS model is capable of (i) modulating the inflammatory response, (ii) influencing neuroprotective mechanisms and (iii) promoting the differentiation of OPCs, to achieve greater preservation of myelin and axonal integrity. Further development of this compound has the merit to be done, while only the clinical setting will validate its therapeutic potential.

## 4. Materials and Methods

### 4.1. Animal and Theiler’s Virus Inoculation

Susceptible female SJL/J mice (Charles River, Barcelona, Spain) were housed under controlled conditions in the Animal Resource Facility at the Cajal Institute, CSIC (Madrid): 12 h light/dark cycle, temperature at 20 °C (±2 °C) and 40–50% relative humidity, with ad libitum access to food and water. Four- to six-week-old mice were inoculated, intracerebrally, in the right cerebral hemisphere with 2 · 10^6^ plaque forming units (pfu) of Daniel’s (DA) TMEV strain diluted on DMEM supplemented with 10% Fetal Calf Serum (FCS). Sham mice (4- to 6-week-old) were injected intracerebrally with 30 µL of commercial DMEM supplemented with 10% Fetal Calf Serum (FCS) as previously described [6].

### 4.2. Treatments and Evaluation of Motor Function

General activity can be an indicator of neurological damage or drug action. The progression of the disease on TMEV infected mice (n = 14) was followed by evaluation of spontaneous motor activity on day 60 and 75 post injection using an activity cage (Activity Monitor System Omnitech Electronics, Inc.; Colombus, OH, USA). Data for horizontal and vertical activities were collected for 10 min. While horizontal activity evaluates the duration of time travelled by the animal, vertical activity measures the total number of bear interruptions in the vertical sensor. The experimental group was also evaluated (n = 11). Sixty days after infection, when pathology was established, half of the TMEV mice were injected i.p. with VP3.15 (10 mg/kg), and the other with the correspondent vehicle, once daily for 15 days. Similar experimental protocol was performed on the Sham mice. VP3.15 was synthesized at the CIB-CSIC laboratories following described procedures [22].

### 4.3. Tissue Processing

Mice were anesthetized by i.p. administration of pentobarbital (EUTANAX^®^, Fatro Ibérica, Barcelona, Spain) and transcardially perfused with saline 0.9%. Spinal cord was extracted by extrusion with saline, fixed overnight in 4% paraformaldehyde (PFA) in 0.1 M phosphate buffer (PB), cryoprotected with a 15% and then 30% solution of sucrose in 0.1 M PB and finally frozen at −80 °C. Transverse thoracic spinal cord cryostat sections (20 µm thick: Leica, Nussloch, Germany) were thaw-mounted on Superfrost^®^Plus slides and were then processed for immunohistochemistry, immunofluorescence or eriochrome cyanine staining.

### 4.4. Eriochrome Cyanine Staining

Myelin staining was performed as described previously [33]. For myelin analysis, the sections were dried for 2 h at RT and for 1.5 h at 37 °C in a slide warmer. The slides were then placed in a container with acetone for 5 min at RT and air-dried for 30 min. The sections were stained in 0.2% eriochrome cyanine (EC) solution for 30 min and differentiated in 5% iron aluminum and borax-ferricyanide for 10 and 5 min, respectively, briefly rinsing under tap and distilled water between each step. Myelin was stained in blue and cell bodies in whitish [34]. Sections were analyzed by acquiring 20× reconstructed images with a color camera of a Zeiss microscope (Leica DM 750) and analyzed using Image J software. The analysis performed represents the percentage of demyelinated area as a function of the total area of white matter (µm2). At least five spinal cord sections were examined per animal.

### 4.5. Immunofluorescence Analysis

Sections were first air-dried for 1 h at room temperature (RT). After several rinses with 0.1 M phosphate buffer (PB), the sections were pre-treated for 15 min with 10% methanol in 0.1 M PB and washed three times with 0.1 M PB for 10 min each time. In the case of myelin basic protein (MBP) labeling, sections were delipidated with a battery of ethanol-increasing concentrations (25%, 75%, 95% and 10%) and later, step-back rehydration for the following steps. Sections were blocked for 1 h at RT in 5% normal donkey serum (EMD Millipore, Burlington, MA, USA) and 0.2% Triton X-100 (Sigma-Aldrich, St. Louis, MI, USA) in PB and then incubated overnight at 4 °C with the primary antibodies (Table 1). After rinsing, sections were incubated with the corresponding fluorescent (1:1000, Invitrogen, Paisley, UK) secondary antibodies for 1 h at RT, rinsed and mounted with coverslips in Fluoromount-G (Southern Biotech, Birmingham, AL, USA). In all cases, cell nuclei were stained with Hoeschst 33342 (10 µg/mL, Sigma-Aldrich). Immunofluorescence pictures were acquired with a confocal SP5 microscope (20 µm z-stack at 0.5 µm intervals, 40× objective). Quantification of myelin basic protein (MBP), neurofilament heavy chain (NFH) and microglia (Iba-1) immunostained area were performed in at least 5 sections per mouse using the Image J software. The total number of cells within the central nervous system (CNS) tissue was assessed using Microscopy Image Analysis software (IMARIS, Oxford, UK), counting cells in 5 sections per mouse, under a user-defined threshold of fluorescence. Quantification of oligodendrocyte precursors are presented as the percentage of PDGFRα^+^Olig2^+^ cells referred to Olig2^+^ while the quantification of mature oligodendrocytes is expressed as the percentage of CC1^+^Olig2^+^ cells referred to Olig2.

### 4.6. Immunohistochemistry Analysis

Spinal cord sections were washed 3 times for 10 min with 0.1 M PB. Endogenous peroxidase activity was inhibited with a 3.3% hydrogen peroxide in methanol, at room temperature and protected from light. The tissue was permeabilized in 0.02% Triton X-100 in PB 0.1 M blocked with 5% animal serum and then incubated overnight at 4 °C in blocking buffer with anti-CD4 primary antibody. After rinsing, the sections were incubated for 1 h with the corresponding biotin-conjugated secondary antibody and then developed with diaminobenzidine (DAB) 0.025% and H_2_O_2_ 0.003% in PB. The tissue was dehydrated, subjecting it to increasing alcohol passes and xylene and finally were covered with DPX^®^ and the coverslip was placed. Analysis was expressed as the number of labeled cells as a function of the total area of white matter (µm^2^). Cells were counted avoiding their overlap and were detected with Ponceau Red (Sigma, Tokyo, Japan, 78376).

### 4.7. Statistical Analysis

The data were expressed as the mean ± SEM and analyzed with Sigma Plot version 11.0 (Systat Software, San Jose, CA, USA) and GraphPad Prism version 5.0 (San Diego, CA, USA). Student’s *t*-test was used to compare pairs of the different groups of mice with a Mann–Whitney U test for non-parametric data, and a two-way ANOVA test was used for multiple comparisons using a Bonferroni post-hoc in the case of non-parametric data. Minimal statistical significance was set at *p* < 0.05: * *p* < 0.05, ** *p* < 0.01, *** *p* < 0.001.

## Figures and Tables

**Table 1:** List of antibodies used in this study.

Antibody	Target	Cellular Location	Dilution	Host Species	Class	Manufacturer	Antibody ID
**MBP**	Myelin	Plasma Membrane	1:500	Rat	Monoclonal clone 12	Biorad	aa 82–87
**NFH**	Neurons/ Axons	Cell Body	1:1000	Rabbit	Polyclonal	Abcam	Ab 8135
**Iba-1**	Microglia	Plasma membrane	1:500	Guinea pig	Polyclonal	Synaptic Systems	234 004
**PDGFRα**	OPCs	Plasma membrane	1:200	Goat	Polyclonal	RD Systems	AF 1062
**CC1**	Mature oligodendrocytes	Cell body	1:200	Mouse	Monoclonal clone CC1	Merck Millipore	OP 80
**Olig2**	Oligodendrocyte lineage	Nucleus	1:200	Rabbit	Polyclonal	Merck Millipore	AB 9610
**CD4**	T cell	Plasma membrane	1:250	Rat	Monoclonal	BD Pharmingen	550278

Abbreviations: MBP = myelin binding protein; NFH = neurofilament heavy polypeptide; PDGFRα = platelet-derived growth factor receptor α.

## Data Availability

The datasets supporting the conclusions of this article are available in the DIGITAL.CSIC repository, https://digital.csic.es/handle/10261/36253, accessed on 15 September 2022.
